# Sacrococcygeal polymelia: A case report and literature review

**DOI:** 10.1016/j.jpra.2026.01.023

**Published:** 2026-01-23

**Authors:** Shiyas Mohammedali, Saif Badran, Sohail J. Quazi, Sara Iskeirjeh, Branavan Sivakumaar

**Affiliations:** a Department of Pediatric Plastic Surgery, Sidra Medicine Hospital, Doha, Qatar; b Department of Plastic Surgery, Hamad Medical Corporation, Doha, Qatar; c College of Medicine and Public Health, Flinders University, Adelaide, Australia; d Department of Plastic Surgery, Great Ormond Street Hospital, London, United Kingdom; e Division of Plastic and Reconstructive Surgery, Washington University School of Medicine, Saint Louis, MO, USA

**Keywords:** Polymelia, Accessory limb, Supernumerary limb, Congenital anomaly, Sacrococcygeal malformation

## Abstract

Polymelia, first described by Macewen in 1877, is a rare congenital disorder characterized by the presence of accessory limbs. This case report describes a 2-month-old infant with sacrococcygeal polymelia and reviews the literature on pathophysiology, clinical presentations, and surgical management. Following the initial assessment, imaging studies, and clinical evaluation, the accessory limb was excised while preserving normal anatomy. Postoperatively, the wounds healed well with acceptable cosmetic results. This case report highlights the importance of multidisciplinary care for the treatment of this rare condition through careful preoperative assessment and precise surgical techniques.

## Introduction

Polymelia is extremely rare in humans, with a worldwide incidence of approximately 1.47/100,00. It is characterized by the presence of accessory limbs that are often underdeveloped or non-functional with variations in size and structure.^1^ This article describes a case of polymelia with an accessory limb attached to the pelvis that was previously reported in a pediatric journal without describing the surgical management. Salameh K, Al-Bedaywi R, Elkabir NA, et al. Duplicate tail-like right lower limb: A rare congenital malformation 2020; 2(2): 1-3.^2^ In this article, we report the surgical approach, intraoperative challenges, and functional outcome for this rare case of polymelia.

## Case report

A 2-month-old female infant presented to the pediatric plastic surgery clinic at Sidra Hospital with an accessory tail-like leg arising from the sacrococcygeal region. The parents reported that the mother had undergone several antenatal ultrasound examinations, however the accessory limb was not detected during the scans, and the infant’s extra limb-like structure was noted only after the uneventful vaginal delivery.

**Figure 1:**
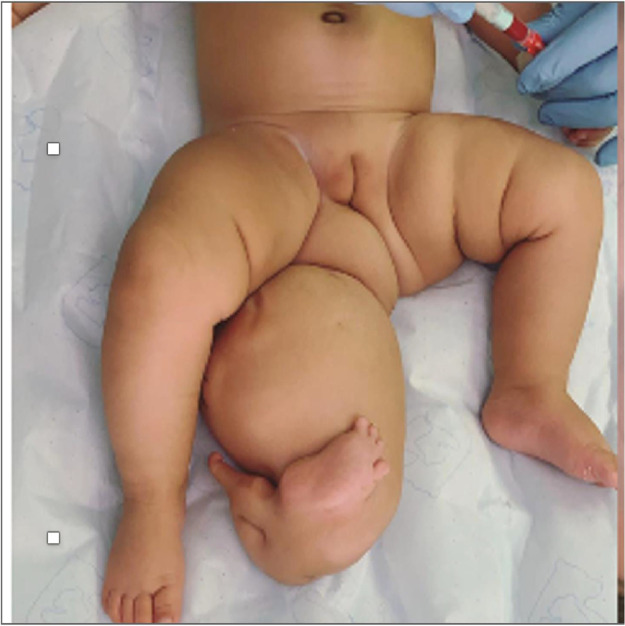
Polymelia anterior view showing accessory limb arising from right side of perineal area, distorting the external genitalia.

Clinical examination revealed a healthy 2-month-old infant (height: 59 cm, weight: 6.26 kg) with an accessory limb near the right ischial tuberosity and lateral to the anal orifice (Figure 1). A small sinus was found at the 5 o’clock position surrounding the right ischial tuberosity. The foot appeared to have two distinct toes owing to its deep first interdigital cleft at the distal end. The parents reported no family history of similar inherited disorders or antenatal exposure to drugs or genotoxic agents.

### Preoperative imaging

**Figure 2:**
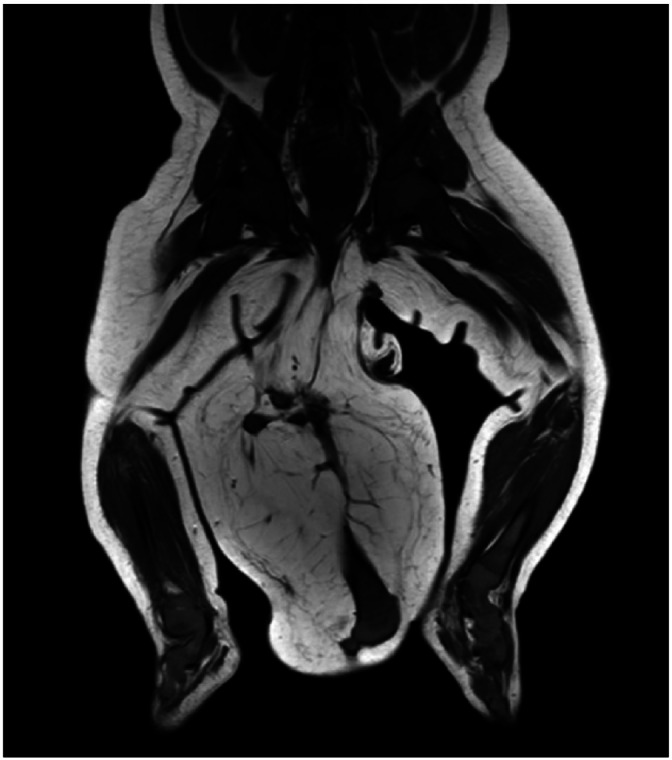
MR how’s narrow origin and close proximity of the accessory limb to the anal verge with malformed hip.

**Figure 3:**
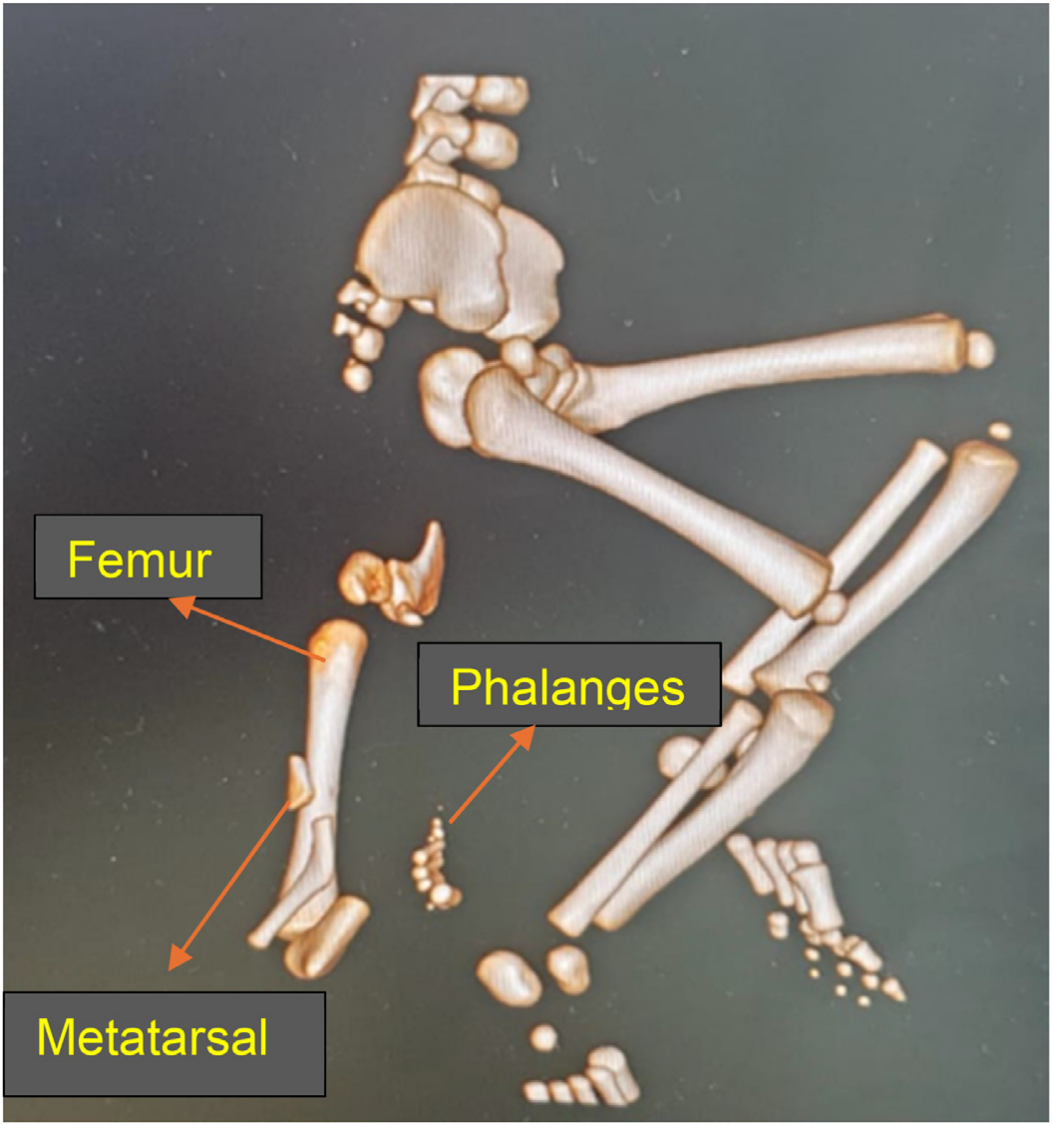
CT image of Extra-limb showing bony elements with fully developed femur and proximal articulation along the partly formed acetabulum. Metatarsal and phalanges of extra-feet also could be seen.

MRI revealed an accessory lower limb originating posteriorly from the right gluteal region, consisting of a malformed hip attached to the tip of the coccyx by a tail-like fibrocartilaginous band (Figure 2). CT Scan showed an accessory femur articulating with the malformed hip proximally and distally with the rudimentary tibia, which terminated in two malformed appendages having 4 rays on the left and a rudimentary ray on the right (Figure 3). Multiple pre- and post-coccygeal cysts were noted, indicating a strong suspicion of sacrococcygeal teratoma.

### Anesthetic and intraoperative management

The infant was assessed preoperatively by a multidisciplinary team of pediatric plastic surgeons, pediatric orthopedic surgeons, anesthesiologists, and radiologists to plan the steps involved in major surgery. The preoperative investigations were normal, and the infant was kept on nothing by mouth for 4 h before the surgery*.*

General anesthesia was induced with sevoflurane in 100% oxygen, and the airway was secured with a cuffed 3.5 endotracheal tube. Anesthesia was maintained with sevoflurane (1–2%), fentanyl, and rocuronium. Continuous ECG, noninvasive blood pressure, pulse oximetry, and end-tidal CO₂ monitoring were used throughout. The infant was positioned prone with meticulous padding.

**Figure 4:**
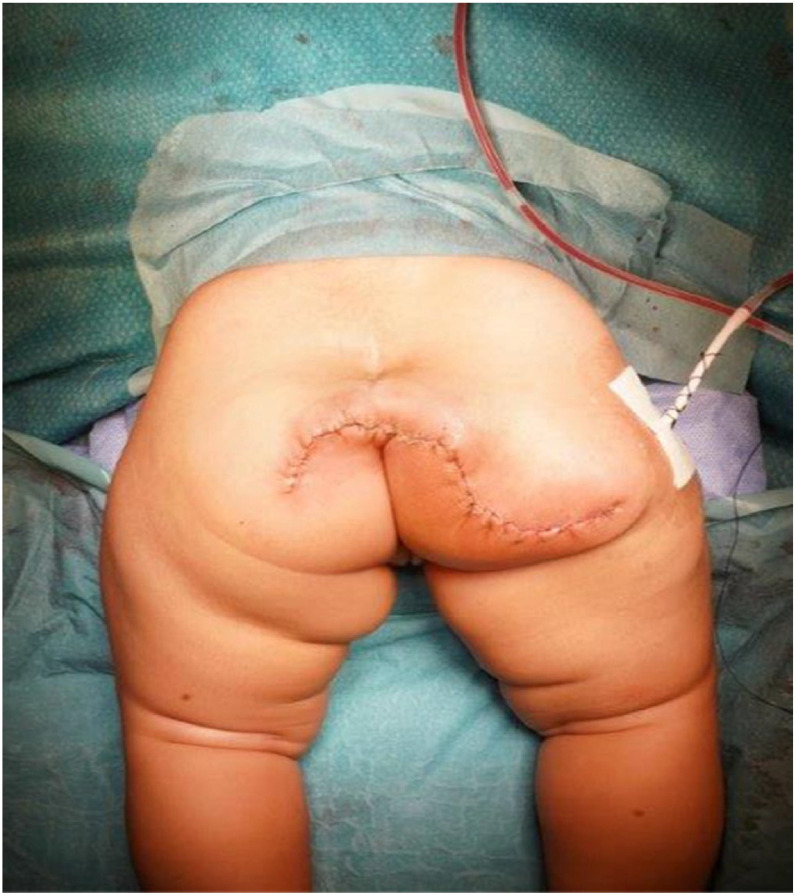
Immediate postoperative image showing the final lazy ‘Z’ buttock scar and largely maintained buttock contour and symmetry.

Intraoperatively, a large limb-like structure was identified originating from the coccyx, with multiple skin folds and significant excess skin at its base. A chevron incision was taken along, the accessory limb which had an enlarged feeding artery arising probably from the aortoiliac bifurcation or the median sacral artery. Careful dissection allowed complete excision of the accessory limb along with the coccyx after ligation of the median sacral vessels. The pelvic floor was reconstructed, and the wound was closed in layers with a drain in situ. Excess skin excision yielded a well-aligned lazy-Z scar. Postoperatively, the infant was observed in the PICU overnight, shifted to the ward on day 1, drains removed on day 3, and discharged on day 7 with satisfactory wound healing (Figure 4).

### Histopathological findings

sohailk7@gmail.com Histopathological analysis of the limb revealed a mass weighing 892 grams, covered by skin and bifurcating into two appendages—one with four toes, and the other a single broad digit. Microscopic examination demonstrated well-differentiated mature adipose tissue with fibrous septa, striated skeletal muscle, myelinated peripheral nerve bundles, and multiple Pacinian corpuscles. The osseous elements contained cartilaginous components with age-appropriate endochondral ossification. Hematoxylin and eosin staining confirmed mature, well-differentiated tissues without immature or malignant elements. No teratomatous components were present, differentiating the lesion from sacrococcygeal teratoma. Immunohistochemistry was not required, as the histomorphology clearly demonstrated mature tissue without atypia.

### Functional outcome

After discharge the infant was followed regularly in the clinic, and the outcomes were assessed using the Functional Status Scale (FSS), a pediatric measure that quantifies function across six domains: mental status, sensory, communication, motor, feeding, and respiratory. Each domain was scored from 1 (normal) to 5 (very severe dysfunction), with a minimum total score of 6 indicating a normal function.

The preoperative FSS was 7, attributed to a motor score of 2 due to the accessory limb. At 3 months, the FSS improved to 6, reflecting age-appropriate development. Examination showed a well-healed scar, unrestricted hip and pelvic motion, and normal anal sphincter tone. At 12 months, the FSS remained 6, with independent walking, normal gait, and no urinary or fecal incontinence.

## Discussion

Polymelia refers to the presence of one or more accessory limbs in various parts of the body.^3^ For clarity and consistency, we refer to the condition as ‘polymelia’ and use ‘accessory limb’ to describe the anatomical anomaly. The differential diagnosis for polymelia includes sacrococcygeal teratoma, fetiform teratoma, and parasitic (pygophagus) twins, which can be distinguished through surgical anatomy, imaging, and histopathology.

Researchers have proposed environmental exposures, embryogenic influences, genetic factors, and mutagenic drugs as probable hypothesis to explain the etiology of polymelia. Embryogenic influences may occur during 4–5 weeks of gestation when several teratogenic events can affect developing limbs through interactions between ectodermal and mesodermal cells. During embryogenesis, the apical Ectodermal Ridge (AER) and the zone of proliferating activity (ZPA) in the limb buds provide reciprocal signals, such as sonic hedgehog (Shh), to regulate limb growth.^4^^,^^5^ Osaki et al. reported that axial limb specification is influenced by specialized mesodermal cells that release a diffusible morphogen; when its concentration is high in more than one region, more than one limb bud may develop.^6^

The diagnosis of polymelia is based on clinical findings and imaging, although these conditions are often missed during antenatal ultrasonography. Plain radiographs can show bony elements such as the femur, acetabulum, metatarsals, and phalanges. Magnetic Resonance Imaging (MRI) provides high-resolution images of soft tissue and can also reveal the origin of the limb with proximity to vital structures (e.g., anal verge) or absence of muscle components in the limb. Three-dimensional Computed Tomography (3D CT) provides a full structure of bony elements and malformations.

Functional outcome is an important factor in the treatment of polymelia. Acharya et al. reported a case in which, despite successful accessory limb removal, the child required serial plasters and soft-tissue release for pre-existing flexion contracture and equinovarus deformity. At 5 years of follow-up, residual abnormalities persisted, including a 5° knee flexion deformity, 10° equinus, and a limb-length discrepancy of 2.5 cm.^7^ Osaki et al. described a rare case of incomplete lower-limb duplication with a tube-like accessory structure extending from the buttock to the back of the thigh. The foot showed severe duplication, with multiple dorsal and plantar toes. The surgeons performed staged functional reconstruction, creating a neurovascular island flap from plantar toes to restore a normal five-toe, plantigrade foot, achieving good running at three-and-a-half-years-old despite residual clubfoot deformity and internal rotation below the knee.^6^

It is essential to measure functional outcome for children with polymelia. We used the Functional Status Scale (FSS), which was developed by health professionals from 11 institutions and found to be an appropriate method to assess functional status in pediatric patients in their study.^8^ In our case, FSS showed normal motor function by 3 months postoperatively. At 12 months, the child demonstrated full developmental milestones with no leg length discrepancy, pelvic instability, or neurological deficits.

## Conclusion

Polymelia is a rare condition that has been reported sporadically in the medical literature. It is most effectively managed with early surgical excision, to achieve good functional and cosmetic outcomes. Due to the anatomical complexity and the involvement of multiple organ systems, a multidisciplinary approach is essential in the management of polymelia.

## Ethical approval

Not required.

## Disclosure

During the preparation of this work, the author(s) used Grammarly and QuillBot to improve language and readability. After using this tool/service, the author(s) reviewed and edited the content as needed and take(s) full responsibility for the content of the publication.

## Funding

None.

## Declaration of competing interest

None declared.
